# Prospective nutrition-inflammation markers for predicting early stoma-related complications in patients with colorectal cancer undergoing enterostomy

**DOI:** 10.3389/fonc.2024.1409503

**Published:** 2024-08-23

**Authors:** Jie Yuan, Fan Jiang, Xiaochao Fu, Yun Hou, Yali Hu, Qishun Yang, Liyang Liu, Yufu Wang, Wangwang Sheng, Fuao Cao, Jinghu He, Guanglei Chen, Cheng Peng, Wei Jiang

**Affiliations:** ^1^ Department of Health Management, Beidaihe Rehabilitation and Recuperation Center of the Chinese People’s Liberation Army, Qinhuangdao, China; ^2^ Department of Colorectal Surgery, Changhai Hospital, Naval Medical University, Shanghai, China; ^3^ Neuroendocrine Department, 72nd Group Army Hospital, Huzhou University, Huzhou, Zhejiang, China; ^4^ Depatrment of General Surgery, Shanghai Rongtong 411 Hospital, Shanghai, China

**Keywords:** stoma complication, colorectal cancer, neutrophil-to-albumin ratio, glucose-to-lymphocyte ratio, predicting marker

## Abstract

**Background:**

Enterostomy is important for radical resection of colorectal cancer (CRC). Nevertheless, the notable occurrence of complications linked to enterostomy results in a reduction in patients’ quality of life and impedes adjuvant therapy. This study sought to forecast early stoma-related complications (ESRCs) by leveraging easily accessible nutrition-inflammation markers in CRC patients.

**Methods:**

This study involved 470 individuals with colorectal cancer who underwent intestinal ostomy at Changhai Hospital Affiliated with Naval Medical University as the internal cohort. Between January 2016 and December 2018, the patients were enrolled and randomly allocated into a primary training group and a secondary validation group, with a ratio of 2:1 being upheld. The research encompassed collecting data on each patient’s clinical and pathological status, along with preoperative laboratory results. Independent risk factors were identified through Lasso regression and multivariate analysis, leading to the development of clinical models represented by a nomogram. The model’s utility was assessed using decision curve analysis, calibration curve, and ROC curve. The final model was validated using an external validation set of 179 individuals from January 2015 to December 2021.

**Results:**

Among the internal cohort, stoma complications were observed in 93 cases. Multivariate regression analysis confirmed that age, stoma site, and elevated markers (Mon, NAR, and GLR) in conjunction with diminished markers (GLB and LMR) independently contributed to an increased risk of ESRCs. The clinical model was established based on these seven factors. The training, internal, and external validation groups exhibited ROC curve areas of 0.839, 0.812, and 0.793, respectively. The calibration curve showed good concordance among the forecasted model with real incidence of ostomy complications. The model displayed outstanding predictive capability and is deemed applicable in clinical settings, as evidenced by Decision Curve Analysis.

**Conclusion:**

This study identified nutrition-inflammation markers (GLB, NAR, and GLR) in combination with demographic data as crucial predictors for forecasting ESRCs in colorectal cancer patients. A novel prognostic model was formulated and validated utilizing these markers.

## Introduction

The establishment of a fecal stoma (colostomy or ileostomy) is warranted in a range of pathological conditions, encompassing both malignant and benign etiologies. Predominantly, stoma formations are integral to the management of colorectal cancer (CRC) (1), a prevalent malignancy ranking third in men and fifth in women on a global scale (2), contributing to approximately 10% of cancer-related mortalities (3). The main goal of ostomy formation is to reduce the likelihood of postoperative complications (4). Nevertheless, the occurrence of postoperative complications related to stomas can be significant, varying between 20-70% according to diverse studies (5–8). Early stoma-related complications (ESRCs), manifesting within 30 days post-surgery with reported incidences ranging from 3-82% (9), can significantly impact the quality of life (10), prolong hospitalization (11), necessitate additional care (12), and impose heightened psychological and financial burdens on patients (13). Furthermore, ESRCs have the potential to delay the initiation of adjuvant therapy, resulting in an unfavorable prognosis (14).

In recent years, serum markers indicative of the nutrition-inflammation status have been employed to anticipate various postoperative complications. An elevated C-reactive protein to albumin ratio (CAR) has demonstrated predictive value for anastomotic complications subsequent to radical resection of gastric cancer (15), CRC (15), and esophageal cancer (16), among other digestive malignancies. Increased preoperative neutrophil-to-lymphocyte ratio (NLR) has been linked to postoperative pancreatic fistula (17) and postoperative appendectomy site infection (18). Standard preoperative laboratory assessments, encompassing routine blood and liver function tests such as serum albumin, lymphocyte count, neutrophil count, C-reactive protein, and other markers, offer insights into a patient’s inflammation and nutritional status, presenting advantages in terms of simplicity and accessibility. Patients with CRC exhibit a higher vulnerability to malnutrition and inflammation compared to individuals with other tumor types, attributed to factors like obstruction, dietary restriction, or malabsorption (19). There is a scarcity of research aimed at forecasting the onset of early postoperative stoma-related complications using preoperative serum nutrition-inflammation markers. This study was conducted to assess the prognostic significance of these markers for early stoma-related complications in CRC patients and establish a clinical model for predicting such complications.

## Patients and methods

### Study population

**Figure 1:**
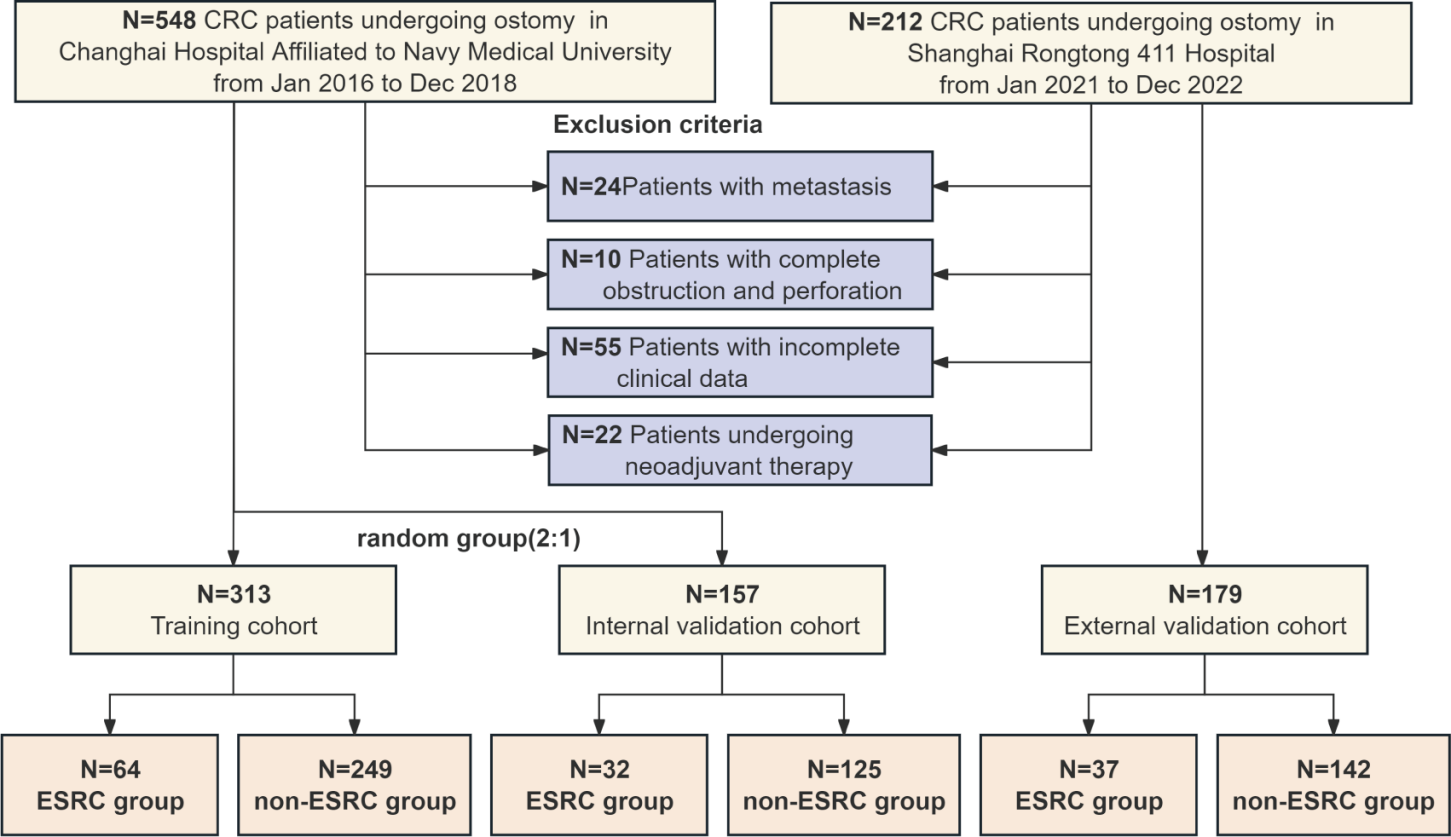
Workflow of study population.

The internal cohort of this study consisted of CRC patients who underwent ileostomy or colostomy at Changhai Hospital Affiliated with Naval Medical University between January 2016 and December 2018. The external cohort comprised CRC patients treated at Shanghai Rongtong 411 Hospital from Jan 2015 to Dec 2021. All patients included in the study were pathologically confirmed to have colorectal cancer (CRC) and underwent surgical procedures conducted by colorectal specialists, with stoma sites identified by senior ostomy nurse practitioners. Patient classification was based on pathology criteria outlined in the 7th edition of the AJCC guidelines. Exclusion criteria encompassed patients with distant metastases, incomplete medical records, those who underwent neoadjuvant therapy, and those who underwent emergency operations. The research protocol followed the ethical guidelines outlined in the Declaration of Helsinki, with explicit informed consent obtained from all participating patients. This study was approved by the ethics committee of Changhai Hospital and Shanghai Rongtong 411 Hospital. Following the application of inclusion criteria, patients in the internal cohort were randomly assigned to two groups for the study: a main training group and a supplementary validation group, with a ratio of two to one being preserved. The process of patient inclusion, exclusion, and grouping was clearly delineated in **Figure 1**.

### Data collection

**Figure 2:**
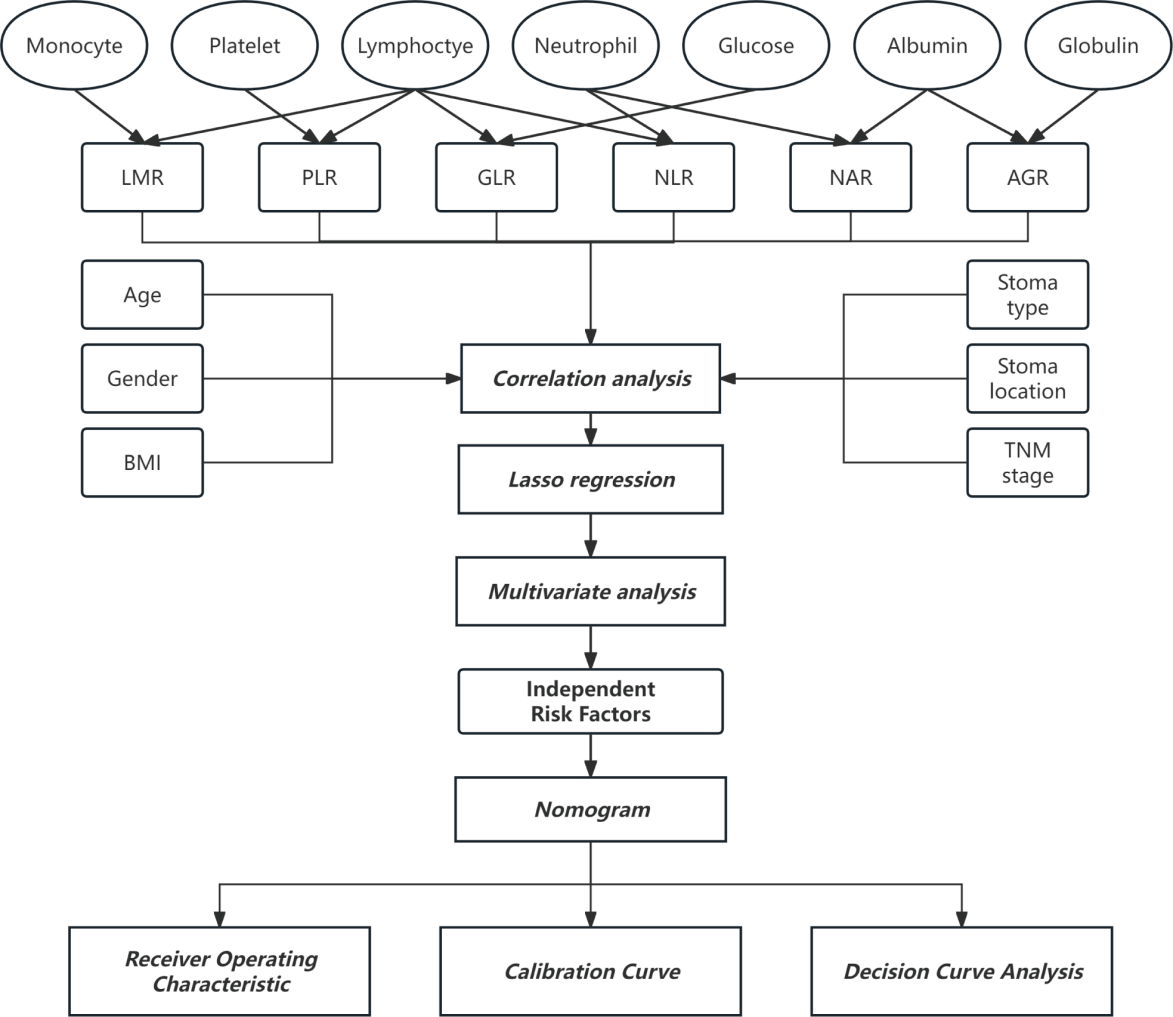
Flow chart of methods.

Demographic and clinicopathological data were gathered, including gender, age, height, weight, tumor stage, stoma type, stoma site, and preoperative peripheral blood parameters. The occurrence of early stoma-related complications (ESRCs) within 30 days post-operation was retrieved from the outpatient and inpatient medical records system. ESRCs encompassed ischemia/necrosis, retraction, bleeding, obstruction, fecal dermatitis, mucocutaneous separation (MCS), and parastomal abscess (20). The Body Mass Index (BMI) was determined by dividing the weight of an individual in kilograms by the square of their height in meters. As depicted in **Figure 2**, the markers of nutrition and inflammation were derived from the analysis of pre-surgical blood samples in the following manner: NLR was obtained by dividing neutrophil numbers by lymphocyte numbers; GLR was calculated by dividing fasting glucose levels by lymphocyte numbers; PLR was determined by dividing platelet numbers by lymphocyte numbers; AGR was computed by dividing the levels of serum albumin by those of serum globulin; LMR was computed by dividing the count of lymphocytes by the count of monocytes; while NAR was determined by dividing the count of neutrophils by the levels of serum albumin.

### Establishment and validation of a nomogram for predicting ESRCs

Lasso regression and multivariate analyses identified independent risk factors among the variables, which were integrated with clinical data to construct a nomogram. A nomogram provides a visual representation of the cumulative impact of various covariates on predicting complications, converting this into a 0-100% probability scale. The “pROC” package was utilized to create ROC curves, facilitating the calculation of the area under the curve (AUC) for assessing model precision. A calibration curve was carried out for assessing the model’s goodness-of-fit, with deviations from the 45° diagonal indicating potential under- or overprediction by the model. Decision curve analysis (DCA) was performed with “rmda” package, which assesses the net benefit across various threshold probabilities to gauge the clinical effectiveness of the nomogram. The study’s procedural flow is depicted in **Figure 2**.

### Statistical analysis

Statistical analyses were conducted through SPSS (version 25.0) and R (version 4.3.0) software. Continuous data were expressed as mean ± standard deviation or median with IQR and analyzed through Mann–Whitney U test or Student’s t-test as applicable. Categorical data were compared utilizing Chi-square test or Fisher’s exact test, with outcomes presented as frequencies and percentages. To ensure objectivity, ROC analysis was employed for determining the specificity of each nutrition-inflammation marker and identify the optimal cut-off value for each continuous variable. Multivariate analysis identified independent risk factors, presented as odds ratios (ORs) with 95% confidence intervals (CIs). The significance of the calibration curves was assessed through Hosmer-Lemeshow test, with p > 0.05 suggesting strong model reliability. All p values were calculated for both tails, with statistical significance set at p < 0.05.

## Results

### Baseline characteristics of the study cohort

Within this research, the internal cohort comprised 548 eligible patients, while the external cohort consisted of 212 individuals. All individuals underwent thorough review using the inpatient record system. Following the application of exclusion criteria, 111 patients were excluded, resulting in 470 eligible patients within internal cohort and 179 within external cohort. Subsequently, the internal cohort was randomly split to a training cohort as well as the internal validation cohort, maintaining a ratio of 2:1. The training group comprised 313 patients, while the internal validation group consisted of 157 patients. Comparative analysis across the groups, encompassing demographic data, stoma conditions, and pathological characteristics, showed no statistically significant variances (**Table 1**), indicating a lack of meaningful disparities among the three groups.

**Table 1:** Baseline of study population.

Variables, n(%)	All cohort	Training cohort	Internal Validation cohort	External validation cohort	p-vale
**all**	649(100)	313(48.23)	157(24.19)	179(27.58)	
**age, yrs**					0.593
** >65**	414(63.79)	205(65.50)	100(63.69)	109(60.89)	
** ≤65**	235(36.21)	108(34.50)	57(36.31)	70(39.11)	
**gender**					0.611
** Male**	347(53.47)	190(60.70)	93(59.24)	64(35.75)	
** Female**	302(46.53)	123(39.30)	64(40.76)	115(64.25)	
**BMI, kg/m**2					0.888
** ≥24**	312(48.07)	153(48.88)	73(46.50)	86(48.04)	
** <24**	337(51.93)	160(51.12)	84(53.50)	93(51.96)	
**stoma type**					0.256
** Loop**	614(94.61)	296(94.57)	152(96.82)	166(92.74)	
** One-end**	35(5.39)	17(5.43)	5(3.18)	13(7.26)	
**Stoma site**					0.599
** ileum**	602(92.76)	288(92.01)	145(92.36)	169(94.41)	
** colon**	47(7.24)	25(7.99)	12(7.64)	10(5.59)	
**TNM stage**					0.821
** I & II**	339(52.23)	167(53.35)	79(50.32)	93(51.96)	
** III**	310(47.77)	146(47.65)	78(49.68)	86(48.04)	
**lymphocyte, 10**9/L	1.58(0.68)	1.57(0.69)	1.60(0.73)	1.60(0.71)	0.520
**neutrophil, 10**9/L	3.53(1.94)	3.45(1.98)	3.64(2.07)	0.44(0.24)	0.233
**monocyte, 10**9/L	0.42(0.19)	0.42(0.19)	0.41(0.21)	3.25(1.88)	0.614
**platelet, 10**9/L	231(97)	237(97)	223(96)	234(95)	0.563
**albumin, g/L**	41(6.0)	41(6.0)	40(6.0)	41(5.0)	0.873
**globulin, g/L**	28(5.0)	28(5.0)	27(5.0)	27(4.5)	0.983
**glucose, mmol/L**	5.30(1.10)	5.30(1.10)	5.30(1.00)	5.3(1.00)	0.692
**NLR**	2.240(1.489)	2.207(1.567)	2.267(1.385)	2.077(2.087)	0.557
**GLR**	3.414(1.880)	3.417(1.807)	3.412(2.061)	3.396(3.395)	0.579
**PLR**	153.97 (87.85)	156.25 (87.51)	146.4 (82.43)	154.63 (81.79)	0.799
**AGR**	1.5(0.348)	1.5(0.34)	1.5(0.37)	3.608(3.602)	0.965
**LMR**	3.702(2.016)	3.729(2.176)	3.667(1.919)	1.517(1.509)	0.535
**NAR**	0.085(0.051)	0.084(0.049)	0.088(0.051)	0.081(0.081)	0.262

BMI, Body Mass Index; NLR, neutrophil to lymphocyte ration; GLR, glucose to lymphocyte ratio; PLR, platelet to lymphocyte ratio; AGR, albumin to globulin ratio; LMR, lymphocyte to monocyte ratio; NAR, neutrophil to albumin ratio.

In the internal cohort, a total of 96 ESRCs were documented. The most prevalent complication was fecal dermatitis, accounting for 61 cases (63.54%), followed by 15 cases of MCS at 15.64%. Other complications included 3 cases of stoma bleeding (3.12%), 3 cases of stoma retraction (3.12%), 4 cases of stoma ischemia/necrosis (4.17%), 5 cases of stoma obstruction (5.21%), as well as 5 cases of parastomal abscess (5.21%) (**Table 2**). The clinicopathological features of the ESRC and non-ESRC groups are outlined in **Table 1**. The incidence of ESRCs exhibited associations with age, gender, stoma site, laboratory parameters, and serum nutrition-inflammatory markers (all P < 0.05, **Table 3**).

**Table 2:** Case of ESRCs in the Internal cohort.

ESRC	Case, n	Percent, %
All case	96	100
Fecal dermatitis	61	63.54
Mucocutaneous separation	15	15.63
Parastomal abscess	5	5.21
Stoma obstruction	5	5.21
Stomach ischemia/necrosis	4	4.17
Stoma retraction	3	3.12
Stoma bleeding	3	3.12

**Table 3:** Comparison of CRC patients with ESRC and non-ESRC in the internal cohort.

Variables, n(%)	Internal cohort	ESRC group	Non-ESRC group	p-value
**All cases**	470(100)	96(20.43)	374(79.57)	
**age, yrs**				***0.003***
** >65**	305(64.9)	50(52.08)	255(68.18)	
** ≤65**	165(35.1)	46(47.92)	119(31.82)	
**gender**				***0.040***
** Male**	283(60.2)	49(51.04)	234(62.57)	
** Female**	187(39.8)	47(49.96)	140(37.43)	
**BMI, kg/m**2				0.117
** ≥24**	226(48.1)	53(55.21)	173(46.26)	
** <24**	244(51.9)	43(44.79)	201(53.74)	
**stoma type**				
** Loop**	448(95.3)	88(91.67)	360(96.26)	0.058
** One-end**	22(4.7)	8(8.33)	14(3.74)	
**Stoma site**				***0.001***
** ileum**	433(92.1)	80(83.33)	353(94.39)	
** colon**	37(7.9)	16(16.67)	21(5.61)	
**TNM stage**				0.331
** I & II**	246(52.3)	46(47.92)	200(53.48)	
** III**	157(47.7)	50(52.08)	174(46.52)	
**lymphocyte, 10**9/L	1.58(0.68)	1.30(0.60)	1.64(0.66)	***<0.001***
**neutrophil, 10**9/L	3.53(1.94)	4.25(2.12)	3.35(1.75)	***<0.001***
**monocyte, 10**9/L	0.42(0.19)	0.47(0.21)	0.41(0.20)	***0.007***
**platelet, 10**9/L	231(97)	237(112)	230(94)	0.353
**albumin, g/L**	41(6)	40(6)	41(6)	***0.008***
**globulin, g/L**	28(5)	27(5)	28(5)	0.753
**glucose, mmol/L**	5.30(1.10)	5.5(1.2)	5.2(1.0)	***0.013***
**NLR**	2.240(1.489)	3.059(1.990)	2.065(1.277)	***<0.001***
**GLR**	3.414(1.880)	4.107(1.962)	3.282(1.690)	***<0.001***
**PLR**	153.97(87.85)	170.46(103.19)	146.71(79.95)	***<0.001***
**AGR**	1.5(0.348)	1.48(0.35)	1.5(0.35)	0.112
**LMR**	3.702(2.016)	2.990(1.439)	3.932(1.970)	***<0.001***
**NAR**	0.085(0.051)	0.105(0.054)	0.081(0.046)	***<0.001***

BMI, Body Mass Index; NLR, neutrophil to lymphocyte ration; GLR, glucose to lymphocyte ratio; PLR, platelet to lymphocyte ratio; AGR, albumin to globulin ratio; LMR, lymphocyte to monocyte ratio; NAR, neutrophil to albumin ratio. When the p-value is below 0.05, it will be shown in bold.

### Cut-off value of the systemic inflammatory markers

**Figure 3:**
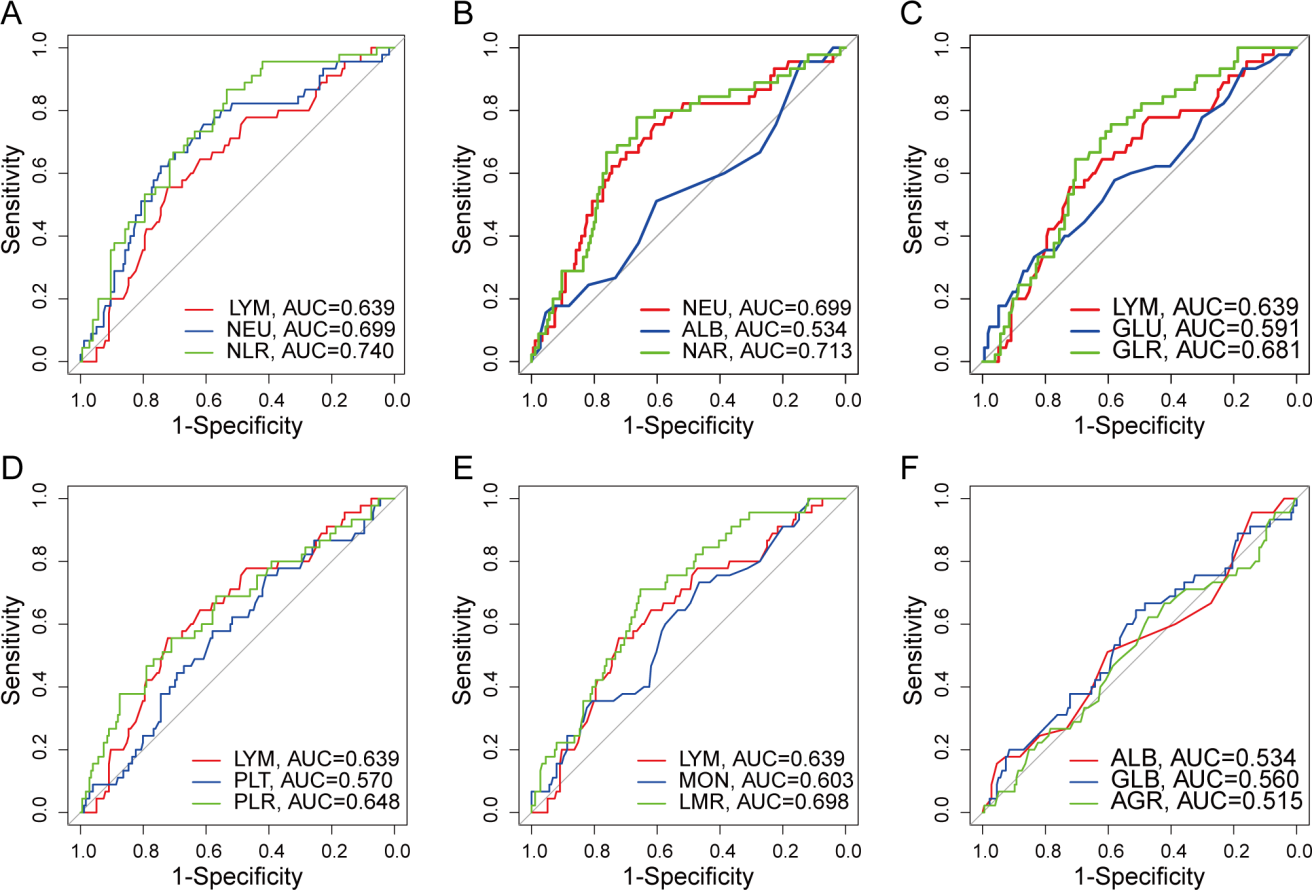
ROC curve of preoperative serum nutrition-inflammation markers. **(A)** ROC curve for NLR. **(B)** ROC curve for NAR. **(C)** ROC curve for GLR. **(D)** ROC curve for PLR. **(E)** ROC curve for LMR. **(F)** ROC curve for AGR.

For the sake of objectivity, the optimal cut-off values for NLR, NAR, GLR, PLR, LMR, and AGR were determined through ROC curve analysis to be 2.422 (AUC, 0.740, **Figure 3A**), 0.086 (AUC, 0.713, **Figure 3B**), 3.662 (AUC, 0.681, **Figure 3C**), 154.297 (AUC, 0.648, **Figure 3D**), 3.546 (AUC, 0.698, **Figure 3E**), and 1.805 (AUC, 0.515, **Figure 3F**), separately.

### Risk factors of ESRCs in patients with CRC

**Figure 4:**
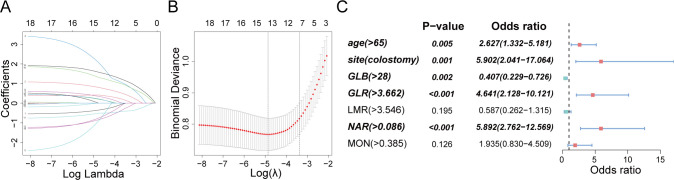
Clinical feature and nutrition-inflammation markers selection using the LASSO regression and multivariate analysis. **(A)** A profile of coefficients was created according to a sequence of logarithmic (lambda) values, with non-zero coefficients emerging from the use of the best lambda. **(B)** The LASSO model’s best lambda parameter was determined through a process of tenfold cross-validation based on the least criterion. The curve depicting the partial likelihood discrepancy (binomial deviation) was charted against the log (lambda). A hypothetical vertical line was added to indicate the optimal lambda, placed at the point of one standard error from the least criterion (the 1-SE rule). **(C)** Graphical representations in the form of forest plots were utilized for the multivariate analysis.

Nineteen candidate parameters (refer to **Table 1**) underwent screening and validation using Lasso regression and five-fold cross-validation according to minimum criteria. Subsequently, 7 potential predictors — age, stoma site, monocyte count, serum globulin, GLR, NAR, and LMR — were selected and included into logistic regression analysis (**Figures 4A, B**). Age (OR 2.627, 95% CI 1.332 – 5.181; p = 0.005), stoma site (OR 5.902; 95% CI 2.041 – 17.064; p = 0.001), and preoperative serum nutrition-inflammation markers — GLB (OR 0.407, 95% CI 0.229 – 0.726; p = 0.002), GLR (OR 4.641; 95% CI 2.128 – 10.121; p < 0.001), and NAR (OR 5.892; 95% CI 2.762 – 12.569; p < 0.001) — were independent risk factors for ESRCs prediction (**Figure 4C**), as indicated by multivariate analysis.

### Development and validation of predictive nomogram

**Figure 5:**
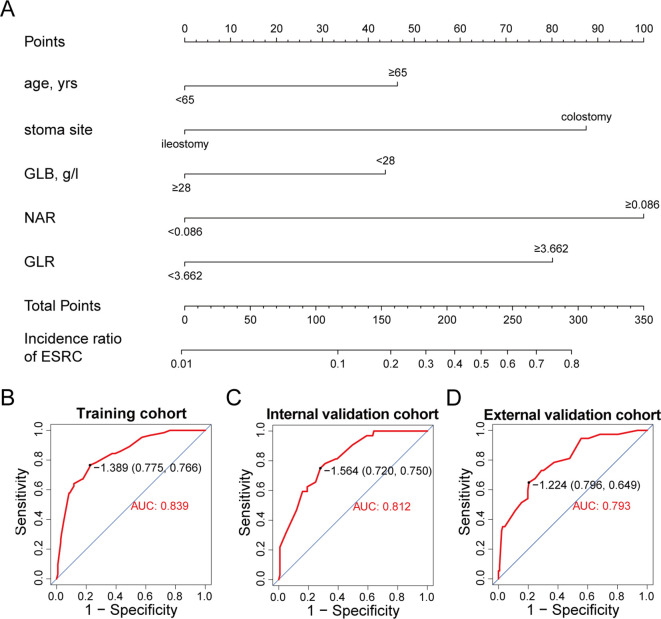
A nomogram for predicting ESRCs. **(A)** nomogram derived from the training group. **(B)** ROC curve for the training group. **(C)** ROC curve for the internal validation group. **(D)** ROC curve for the external validation group.

These identified independent risk factors were integrated into a clinical prediction model for ESRCs, which was represented graphically through a nomogram (refer to **Figure 5A**). In the nomogram, the score allocated to each variable was determined by drawing a vertical line upwards corresponding to its specific value. The cumulative score was calculated through summing the scores assigned to each variable, with the model predicting the likelihood of ESRCs corresponding to the total score expressed as a percentage.

**Figure 6:**
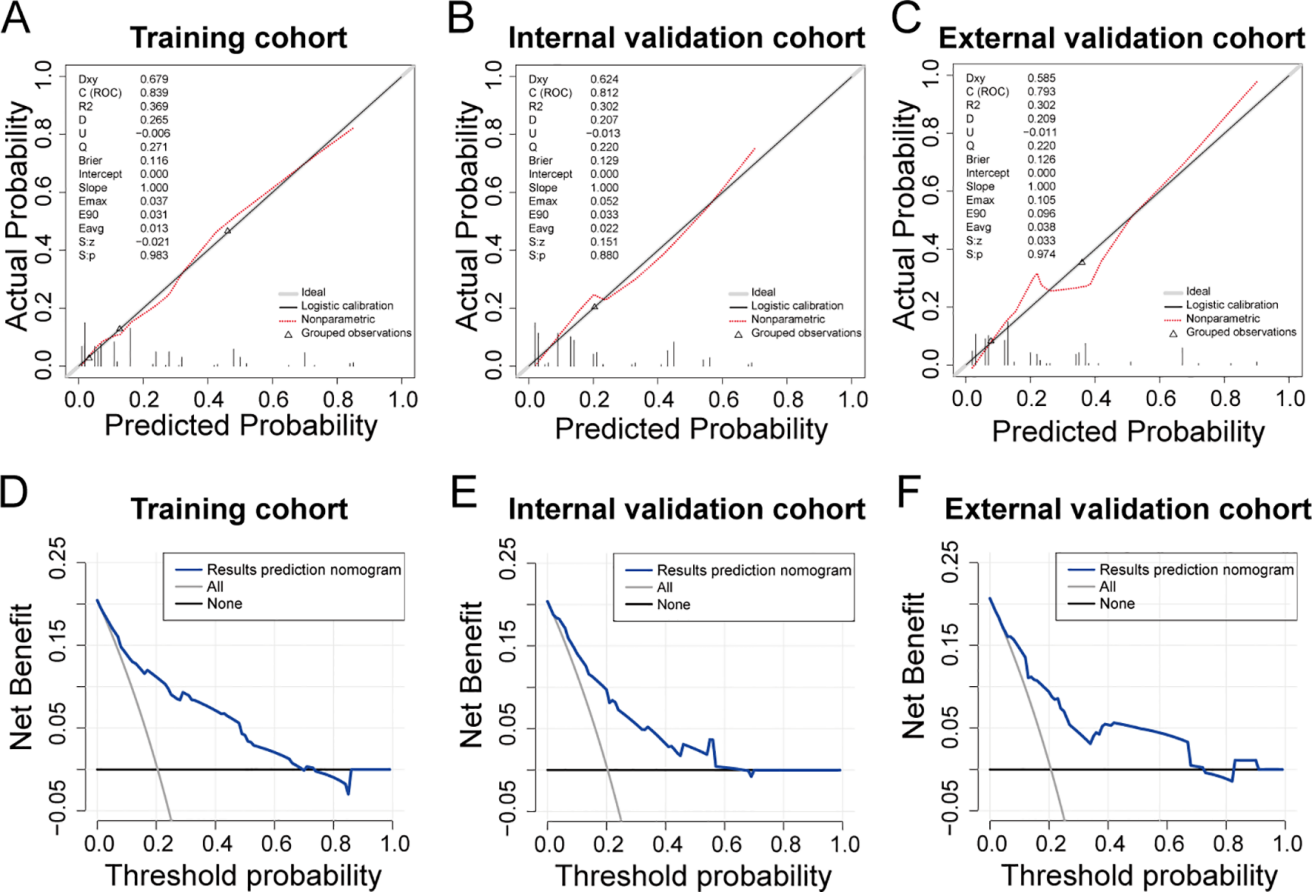
Evaluation of predictive models for ESRCs in patients with CRC. **(A–C)** Calibration curve for the predictive model of the training group **(A)**, the internal validation group **(B)**, and the external validation **(C)**. **(D–F)** DCA curve for the predictive model of the training group **(D)**, the internal validation group **(E)**, and the external validation group **(F)**.

The clinical model exhibited an area under the ROC curve of 0.839 for the training cohort (refer to **Figure 5B**), 0.812 for the internal validation cohort (refer to **Figure 5C**), and 0.793 for the external validation cohort (refer to **Figure 5D**). These values indicate that the predictive model demonstrated strong discriminative capability. Calibration curves (refer to **Figures 6A-C**) illustrated the alignment between the model’s predictions and the actual incidence rates of complications, affirming the model’s reliability. DCA curves depicted the threshold probabilities of the predictive model within the training cohort (refer to **Figure 6D**) and the two validation cohorts (refer to **Figures 6E, F**), providing a comprehensive evaluation of the nomogram’s clinical utility. These analyses highlighted the superior predictive ability of the model, underscoring its potential for practical application in clinical settings.

## Discussion

A stoma holds a critical state within surgical management of colorectal cancer (CRC). Its primary objective is to mitigate the risk of severe complications, notably anastomotic leakage, a significant postoperative concern in CRC cases. Prophylactic ileostomy is advocated for patients with mid to low rectal cancer to avert systemic infections stemming from anastomotic leakage (21). Conversely, colostomy is frequently necessitated in abdominoperineal resection for anal cancer. Individuals presenting with acute conditions like obstruction or perforation may require stoma creation; obstruction affects around 15-30% of CRC patients, while perforation is observed in 1-10% of cases (22–24). An ostomy can offer advantages to patients by reducing surgical trauma, lowering the occurrence of anastomotic complications, and facilitating prompt initiation of adjuvant treatment. Nonetheless, in a vulnerable subset of the population, the potential risks associated with stoma-related complications may outweigh the benefits of fecal diversion (25). Apart from the adverse psychosocial implications of fecal diversion on patients, stoma-related complications, with reported incidence rates of 26.5% (ranging from 2-100%) across diverse stoma types in literature (26), significantly impact the patient’s quality of life as well as body image. This, in turn, results in reduced social engagement and heightened levels of depression and anxiety (27). Hence, the precise recognition of patients prone to stoma-related complications is crucial for enhancing clinical outcomes.

The present study revealed that approximately 20% of patients experienced ESRCs. Peristomal skin issues were the most prevalent, followed by MCS. This investigation encompassed an evaluation of clinicopathological parameters, preoperative laboratory data, and peripheral blood-derived markers as variables. A significant correlation was observed between ESRCs and both clinicopathological factors and preoperative serum nutrition-inflammation markers. While factors such as surgical technique and stoma placement contribute to the occurrence of stoma-related complications, prior research has frequently highlighted the influence of gender, age, and stoma site. The preoperative condition of the patient, encompassing comorbidities, infection, and malnutrition, emerges as a crucial determinant in this context (28–30). Markers included in this study (AGR, NAR, LMR, PLR, NLR, and GLR), calculated using peripheral blood indices, provided more precise indications of the body’s nutrition-inflammation status and the incidence of ESRCs. Furthermore, multivariate analyses revealed that age exceeding 65 years, colostomy, and the presence of three preoperative abnormal markers (NAR > 0.086, GLR > 3.662, and GLB ≤ 28g/L) were identified as independent risk factors for ESRCs.

It is expected that nutrition-inflammation markers could function as predictors of ESRCs. Patients with CRC, particularly those experiencing diminished intake, malabsorption, and local or systemic infections resulting from obstruction or perforation, are prone to malnutrition (19) and systemic inflammatory reactions. Roughly 35% of patients demonstrate moderate to severe malnutrition preoperatively (31). Malnutrition can induce immunosuppression, heightened postoperative infection risk and inflammatory responses, hampered tissue healing, and prolonged recovery periods for gastrointestinal function and hospitalization duration (31, 32). The preoperative inflammatory condition increases patients’ susceptibility to infections, impedes tissue healing, and elevates the likelihood of postoperative complications (33–35). GLB, NAR, and GLR serve not only as nutritional status indicators but also as novel markers of systemic inflammation and disease severity.

The combination of the aforementioned three serum markers, stoma site, and age—factors previously identified as predictive of stoma-related complications (36)—was utilized to develop a novel predictive model for ESRCs. Subsequently, the accuracy and feasibility of this model were validated in an independent cohort. Numerous studies have focused on assessing preoperative nutritional and inflammatory statuses to forecast clinical outcomes in patients with CRC. The application of the Malnutrition Universal Screening Tool (MUST) has been instrumental in evaluating the preoperative nutritional status of CRC patients, demonstrating a correlation between malnutrition, prolonged hospitalization, and unfavorable post-surgical prognosis (37). The assessment of both nutritional status and inflammatory response has been conducted using the Systemic Inflammatory Grade (SIG), which has been established as an independent risk factor for postoperative complications (38). Preoperative inflammatory responses may be elevated in colorectal cancer (CRC) patients experiencing anastomotic leakage, as indicated by increased levels of serum C-X-C Motif Chemokine Ligand 6 and C-C Motif Chemokine Ligand 11 in individuals with rectal cancer and heightened levels of serum high-sensitivity CRP in those with colon cancer (39). Nevertheless, the practical application of these markers and tools in clinical settings is constrained by the intricacy involved in their detection and computation. Some studies have developed nutrition-inflammation markers based on laboratory data, such as CAR (40), NLR (41), LMR (42), AGR (43). These markers have demonstrated their significance in forecasting the clinical outcomes of CRC patients. However, the emphasis of prior studies has predominantly centered on prognosis and intraperitoneal complications, with limited exploration of the association between preoperative nutritional, inflammatory status, and stoma complications. In contrast, the ESRC prediction model developed in this study relies on blood-derived markers, rendering our model convenient and easily accessible without imposing additional burdens on patients.

However, there are various limitations in this study. Primarily, being retrospective in nature, further prospective studies are essential to confirm the predictive relevance of these markers. Secondly, certain factors, including the utilization of support rods, emergency surgical procedures, and the application of immunosuppressive agents, were not taken into account due to the restricted data available. Furthermore, owing to the specialized colorectal surgery and stoma care provided at our institution, some complications were only present in a limited number of cases; hence, all categories of early stoma-related complications were collectively analyzed in this study.

## Conclusion

In conclusion, this study has identified preoperative nutrition-inflammation markers as autonomous risk factors for ESRCs in CRC patients. Additionally, we have formulated a clinical model encompassing NAR, GLR, and clinical parameters, facilitating precise prognostication of ESRC incidence. This innovative nomogram holds substantial clinical utility for effectively stratifying high-risk patients and enabling timely interventions.

## Data Availability

The raw data supporting the conclusions of this article will be made available by the authors, without undue reservation.
